# Proteasome inhibition as a therapeutic approach in atypical teratoid/rhabdoid tumors

**DOI:** 10.1093/noajnl/vdaa051

**Published:** 2020-04-14

**Authors:** Andrew Morin, Caroline Soane, Angela Pierce, Bridget Sanford, Kenneth L Jones, Michele Crespo, Shadi Zahedi, Rajeev Vibhakar, Jean M Mulcahy Levy

**Affiliations:** 1 Department of Pediatrics, University of Colorado School of Medicine, Aurora, Colorado; 2 Department of Pharmacology, University of Colorado School of Medicine, Aurora, Colorado; 3 Morgan Adams Foundation Pediatric Brain Tumor Research Program, Aurora, Colorado

**Keywords:** drug screen, marizomib, proteasome, rhabdoid tumors

## Abstract

**Background:**

Atypical teratoid/thabdoid tumor (AT/RT) remains a difficult-to-treat tumor with a 5-year overall survival rate of 15%–45%. Proteasome inhibition has recently been opened as an avenue for cancer treatment with the FDA approval of bortezomib (BTZ) in 2003 and carfilzomib (CFZ) in 2012. The aim of this study was to identify and characterize a pre-approved targeted therapy with potential for clinical trials in AT/RT.

**Methods:**

We performed a drug screen using a panel of 134 FDA-approved drugs in 3 AT/RT cell lines. Follow-on in vitro studies used 6 cell lines and patient-derived short-term cultures to characterize selected drug interactions with AT/RT. In vivo efficacy was evaluated using patient derived xenografts in an intracranial murine model.

**Results:**

BTZ and CFZ are highly effective in vitro, producing some of the strongest growth-inhibition responses of the evaluated 134-drug panel. Marizomib (MRZ), a proteasome inhibitor known to pass the blood–brain barrier (BBB), also strongly inhibits AT/RT proteasomes and generates rapid cell death at clinically achievable doses in established cell lines and freshly patient-derived tumor lines. MRZ also significantly extends survival in an intracranial mouse model of AT/RT.

**Conclusions:**

MRZ is a newer proteasome inhibitor that has been shown to cross the BBB and is already in phase II clinical trials for adult high-grade glioma (NCT NCT02330562 and NCT02903069). MRZ strongly inhibits AT/RT cell growth both in vitro and in vivo via a moderately well-characterized mechanism and has direct translational potential for patients with AT/RT.

**Key Points**

- Proteasome inhibition is superior to current methods of killing AT/RT cells in vitro.
- Marizomib is capable of entering the brain to inhibit AT/RT tumors in vivo and improving survival.
- Marizomib is translationally viable for treatment of pediatric AT/RT.

**Importance of the Study**

Atypical teratoid/rhabdoid tumors (AT/RT) of the central nervous system are a rare but aggressive malignancy most commonly occurring within the first 3 years of life. AT/RT patients face a dismal prognosis despite aggressive therapy, with recent efforts focusing on a high-dose chemotherapy approach. Proteasome inhibitors have recently demonstrated success as a targeted therapy in the treatment of acute leukemia (both lymphoblastic and myeloblastic), and the proteasome inhibitor marizomib (MRZ) is currently in Phase II trials in adult glioblastoma. We have determined that AT/RT cell lines and patient-derived cultures are especially susceptible to MRZ in vitro when compared with other currently-approved drugs and that MRZ is capable of reducing tumor growth in an intracranial in vivo model. These results suggest that proteasome inhibition by MRZ is translationally viable for clinical trials as a therapy for AT/RT.

Atypical teratoid/thabdoid tumor (AT/RT) of the central nervous system (CNS) is a rarely occurring but highly aggressive cancer, most commonly appearing within the first 3 years of life. It comprises approximately 2%–3% of all childhood CNS cancers,^1^ but is the single most common malignant CNS tumor to appear before the age of 6 months, and there is no widely accepted standard of care.^2^ Although gross total resection of the tumor and radiotherapy are both associated with improved survival,^3,4^ there is currently no standard chemotherapy.^2^ Complete resection may also be impossible, as AT/RT tumors can appear in nearly any region of the CNS, and up to a fifth of tumors may already have disseminated by the time of diagnosis.^4^ Most AT/RT clinical trials thus far have used nontargeted high-dose chemotherapy (HDC) courses, and although these show some improvement in survival rates over legacy chemotherapies such as anthracyclines,^5^ outcomes remain poor even on these intensive programs (15%–45% 2-year overall survival), and there is a pressing need for new and preferably targeted therapies.

AT/RT tumors are characterized by the loss of SWI/SNF complex tumor-suppressor activity,^6^ which is nearly always caused by inactivation of the core complex component *SMARCB1*^7^ (or, less commonly, *SMARCA4*).^8^*SMARCB1* loss may be caused by deleterious mutations within the gene itself or by alterations or deletions of chromosome 22q11. These mutations are of germline origin in around 25%–35% of patients.^9^ The SWI/SNF chromatin-remodeling complex acts as a master epigenetic regulator, and inactivation through SMARCB1 loss leads to a variety of transcriptional programs eventually leading to tumorigenesis.^8^ As a result of this nonlocalized activity, and the poorly understood differences between the 3 epigenetic/transcriptional subgroups into which AT/RTs are typically divided,^10,11^ it has been difficult to generate targeted therapies. Targeted therapies have begun to emerge, with inhibitors of Aurora Kinase A (NCT02114229), CDK4/6 (NCT03434262), EZH2 (NCT02601937), and several others entering clinical trials, but the field remains profoundly lacking in new approaches when compared with advances in other tumor types.

Along with autophagy, the ubiquitin-proteasome system is a primary mechanism by which cells maintain protein homeostasis and clear misfolded proteins.^12^ Inhibition of the proteasome leads to a buildup of unfolded/misfolded proteins and the accumulation of proteins which require proteasomal turnover for maintenance of proper expression/activity levels.^13^ Several proteasome inhibitors have seen widespread use in the treatment of multiple myeloma and mantle-cell lymphoma^14,15^ and are under investigation for a wide array of solid tumors as well.^14,16^ Marizomib (MRZ) is a recently developed small-molecule proteasome inhibitor, capable of irreversibly binding to and inactivating all 3 enzymatic functions (trypsin-like, chymotrypsin-like, and caspase-like)^17,18^ of both the standard proteasome and the immunoproteasome.^19^ Unlike bortezomib (BTZ) and carfilzomib (CFZ), MRZ is capable of crossing the blood-brain barrier (BBB),^20,21^ at least in the context of a potentially disrupted cancerous BBB,^22^ and is capable of overcoming existing resistance to BTZ or CFZ.^23^

Proteasome inhibitors are capable of either directly inducing cancer cell death or sensitizing cancer cells to apoptosis, by a mechanism currently hypothesized to be an increase in intracellular reactive oxygen species (ROS).^18,24^ MRZ is now featured in several trials for treatment of adult glioblastoma and has shown potential to generate at least partial responses (NCT02903069). In light of this potential, we have investigated the use of MRZ as a putative therapeutic avenue for the treatment of pediatric AT/RT.

## Methods

### Study Approval

Primary patient samples were obtained from Children’s Hospital Colorado and collected in accordance with local and Federal human research protection guidelines and institutional review board regulations (COMIRB 95–500). Informed consent was obtained for all specimens collected.

### Cell Culture

The BT12 and BT16 lines were a gift from the laboratory of Dr Peter Houghton at St. Jude Children’s Hospital. The MAF-737A, MAF-1337A, and MAF-1298A patient-derived cultures were grown from tumor tissue removed from patients either during biopsy, surgical resection, or autopsy at Children’s Hospital Colorado. These cultures were grown for at least 3 passages prior to experimental use and verified as AT/RT cultures by western blot for the loss of *INI1*. The CHLA-266 line was a gift from the laboratory of Dr Annie Huang at The Hospital for Sick Children. All cell lines were routinely verified as free of mycoplasma by the MycoAlert Detection Kit (Lonza) throughout the course of the project, and authenticated by short tandem repeat profiling by the Barbara Davis Center Molecular Biology Service Center both prior to, and toward the conclusion of the experiments. Cell lines were typed into the AT/RT subgroups by RNA sequencing, which is deposited in NCBI’s Gene Expression Omnibus (Edgar et al., 2002) and accessible through GEO Series accession number GSE137777 (https://www.ncbi.nlm.nih.gov/geo/query/acc.cgi?acc=GSE137777). Cells were maintained in a humidified incubator at 37C° with 5% CO_2_. BT12, BT16, and MAF-737A lines were grown in RPMI 1640 media supplemented with 10% Fetal bovine serum (FBS) and 1% penicillin/streptomycin. MAF-1298A and MAF-1337A lines were grown in OptiMEM media supplemented with 15% FBS and 1% penicillin/streptomycin. The CHLA-266 line was grown in Iscove Modified Dulbecco medium supplemented with 20% FBS, 4 mM l-glutamine, 5 µg/mL Insulin, transferrin, selenite, and 1% penicillin/streptomycin. All neurosphere assays were conducted using complete NeuroCult NS-A Proliferation media (STEMCELL Technologies).

### Reagents

The Approved Oncology Drugs Set VIII screening panel was obtained from the NCI-Chemotherapeutic Agents Repository, through Fisher Bioservices. Marizomib was obtained from Adipogen. N-acetyl cysteine (NaC) and Q-VD-OPh were obtained from Sigma–Aldrich. Carfilzomib was obtained from Selleckchem. Chloroquine diphosphate was obtained from Sigma–Aldrich.

### Growth/Viability Assays

For the Approved Oncology Drugs screen, MAF-737A, BT12, and BT16 cells were seeded in 96-well plates at 1000 cell/well in 100 µL and assessed using the CellTiter Glo Luminescent Cell Viability Assay (Promega) after 5 days. For death/rescue assays, cells were plated at 2000 cell/well in 100 µL, and caspase activity and cell death were recorded in 96-well plates on the Incucyte ZOOM platform (Essen BioScience), using the CellEvent Caspase 3/7 detection Reagent (Invitrogen) at 5 µM and YOYO-3 Iodide (Invitrogen) at 200 nM. Cell viability was measured via CellTiter Glo and by measurements of confluence by the Incucyte ZOOM. Neurosphere size was quantified using the Incucyte S3 platform, 7 and 14 days after seeding 1000 cells/well in ultra-low attachment 96-well plates and treating with either MRZ or NaC. For long-term growth assays, cells were seeded at 200–500 cells/well in 1 mL media 12-well plates and grown over 10–14 days with drug administered in fresh media twice weekly (4 total doses), then simultaneously fixed and stained in 1:3 methanol:crystal violet for 15 minutes. Stained cells were washed and then quantified by dissolution in 33% acetic acid followed by spectrophotometric measurement of the dissolved crystal violet.

### Quantitative RT-PCR

Cells were treated with 100 nM MRZ, and RNA was collected at 24 hrs later using the Zymo Quick-RNA MiniPrep Plus kit (Zymo Research), and 2-step Q-RT-PCR conducted using the iScript cDNA Synthesis kit (Biorad) and iTaq Universal SYBR Green Supermix kit (Biorad). Results were normalized to a GAPDH control. Primers used were SOD (CAGGTCCTCACTTTAATCCTCTATC, CATCGGCCACACCATCTT), HSP90 (GGAGATAAACCCTGA CCATTCC, GACAGGAGCGCAGTTTCATA), ATF4 (CTCTTACT GGTGAGTGCAAAGA, TGCGGACCTCTTCTATCAAATC), CHOP (CATGAACAATTGGGAGCATCAG, GGGTCACA TCATTGGCACTA), BiP (TGTTCAACCAATTATCAGCAAACTC, TTCTGCTGTATCCTCTTCACCAGT), spliced XPB1 (CTGAG TCCGAATCAGGTGCAG, ATCCATGGGGAGATGTTCTGG), and total XBP1 (TGGCCGGGTCTGCTGAGTCCG, ATCCA TGGGGAGATGTTCTGG).

### Proteasome Activity Assays

Cells were seeded in 96-well plates, treated with 100 nM MRZ and/or 10 mM NaC for either 6 or 24 hours, and then proteasome activity assayed using the Cell-Based Proteasome-Glo Chymotrypsin-like kit (Promega).

### Flow Cytometry

Cells were treated with MRZ for 24 hours. Prior to collection, the cells were stained for superoxide and general oxidative stress using the Cellular ROS/Superoxide Detection Assay Kit (Abcam). Stained cells were then assayed by flow cytometry on the Gallios 561 (Beckman Coulter), and the Kaluza acquisition software (Beckman Coulter).

### Radiation Treatment

Cells were seeded in 96-well plates, and irradiated using a Cs-137 source for dosages calculated to 0, 0.5, 1, 2, or 4 Gy. MRZ doses were added immediately following irradiation. The results were assessed using CellTiter Glo.

### MRZ Penetration of the Cerebellum

Eight 6-week-old athymic nude mice were intraperitoneally injected with either 200 µg/kg MRZ or an equivalent volume of vehicle (2% Dimethyl sulfoxide [DMSO] in saline) and sacrificed 1 hour later. The cerebellum of each mouse was extracted, mechanically disaggregated, and passed through a 70-µM cell filter. Cells were then aliquoted at 10k cell/well of a 96-well plate and assayed using the Cell-Based Proteasome-Glo Chymotrypsin-like kit (Promega).

### Intracranial Xenograft Models

Luciferase-expressing MAF-737A or BT16 cells were injected via intracranial guidescrew into the cerebellums of 6-week-old athymic nude mice at 500k cells/mouse (MAF-737A) or 50k cells/mouse (BT16), and permitted to grow for 14 or 21 days post-tumor injection. At that point, twice-weekly intraperitoneal injection with either MRZ or an equivalent volume of vehicle (2% DMSO in saline) was initiated for mice with confirmed tumor formation and continued until sacrifice. BT16-transplanted mice received 150 µg/kg MRZ per dose, and MAF-737A-transplanted mice received 200 µg/kg. Upon sacrifice, the cerebellums were collected for histological analysis and tumor cell re-isolation. Survival was assessed from the time of initial treatment. All experimental procedures were approved by the Office of the Institutional Animal Care and Use Committee at the University of Colorado (IACUC protocol 00052).

### Western Blot

Protein samples were collected in stringent RIPA buffer and resolved by sodium dodecyl sulfate–polyacrylamide gel electrophoresis. Target proteins were blotted using Cell Signaling antibodies against Poly (ADP-ribose) polymerase (PARP) (#9542), caspase 3 (#9662), cleaved caspase 3 (#9661), and GAPDH (#5174). Visualization was accomplished using a Cell Signaling secondary HRP-conjugated anti-rabbit (#7074) and G:Box imager (Syngene).

### Statistical Analysis

All statistics are presented as the mean ± standard error of the mean. Student’s *t*-tests, Dunnett’s multiple comparisons tests, and ANOVA were performed using Graphpad Prism software version 7, using data collected from at least 3 biological replicates/experiment as appropriate. For in vitro experiments, 3 technical replicates were executed per biological replicate (totaling 9 observations). Significance was determined at *P* ≤ .05. Tumor RNA sequencing data for subtyping was visualized using ClustVis.^25^

## Results

### Proteasome Inhibition Is Strongly Inhibitory Toward AT/RT Growth In Vitro

**Figure 1.**
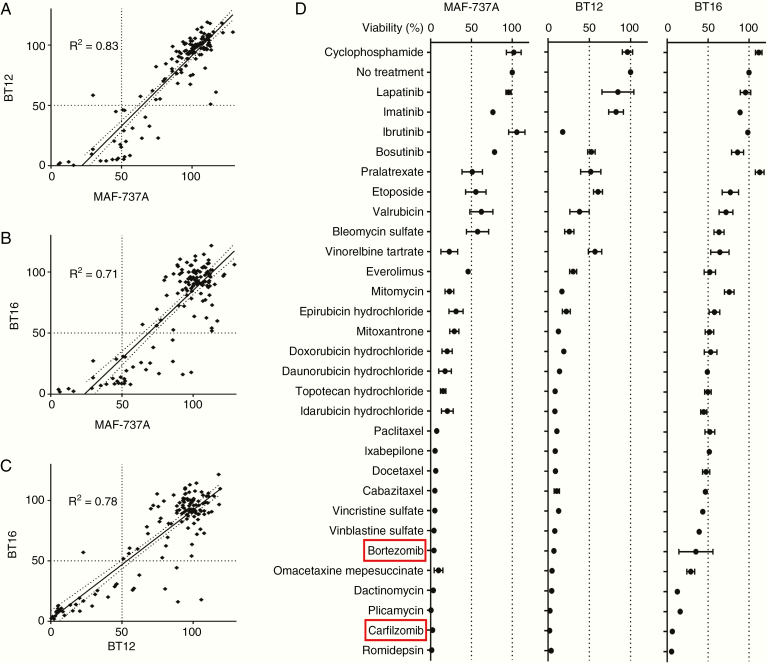
Proteasome inhibitors strongly inhibit the growth of atypical teratoid/thabdoid tumor (AT/RT) cell lines in vitro. (A–C) Screening results from a 134-drug panel in 3 AT/RT cell lines, displayed as percent cell viability by CellTiter Glo compared with Dimethyl sulfoxide (DMSO) control. Results from 3 cell lines are then correlated with each other. (D) Cell viability by CellTiter Glo is displayed as a percentage of the DMSO (no treatment) control for 30 compounds selected from the most inhibitory subset of the full 134-drug panel. Cells were assayed after 5 days of 100 nM drug. Error bars indicate the standard error of the mean, *n* = 3.

We initially screened 3 AT/RT tumor cell lines (MAF-737A, BT12, BT16) using a 134-drug panel of compounds that are currently FDA-approved for use in oncology (Supplementary Table S1). The majority of the panel compounds had no significant effect on cell growth when treated at either 1 µM or 100 nM for 5 days, and there was a high degree of correlation in response between the 3 cell lines (Figure 1A–C), indicating that the observed responses are likely reflective of general AT/RT biology, rather than cell line-dependent. Interestingly, in these experimental conditions, many of the drugs currently used in conventional HDC for treatment of AT/RTs (cyclophosphamide, vincristine, doxorubicin, idarubicin, methotrexate, etoposide)^26^ displayed either a mild antiproliferative effect or no effect (Figure 1D), a finding in keeping with the dismal survival rates for these tumors. Conversely, other drugs under recent investigation as strong therapy candidates (various Histone deacetylase inhibitors and some anthracyclines) were comparatively effective, with the proteasome inhibitors BTZ and CFZ standing out as particularly potent. We therefore chose proteasome inhibition as a mechanism for further investigation.

### MRZ Is Broadly Cytotoxic Toward AT/RT Cell Lines Adherently and in 3D Culture

**Figure 2.**
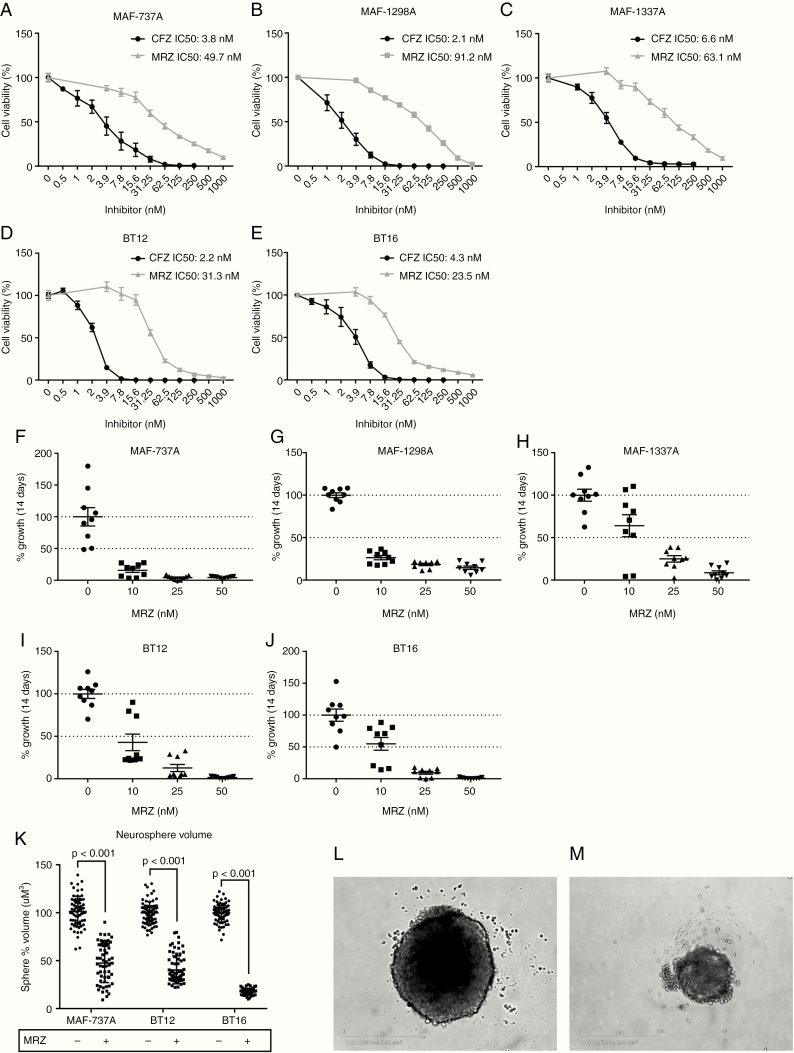
Marizomib (MRZ) inhibits atypical teratoid/thabdoid tumor (AT/RT) cells at clinically achievable doses and in 3D culture. (A–E) Dose response curves to 5-d treatment with either MRZ or CFZ for MAF-737A (A), MAF-1298A (B), MAF-1337A (C), BT12 (D), and BT16 (E) cell lines, by CellTiter-Glo. Error bars indicate the standard error of the mean. (F–J) Cell viability after dosing with MRZ twice weekly for 14 days at 0, 10, 25, and 50 nM MRZ for MAF-737A (F), MAF-1298A (G), MAF-1337A (H), BT12 (I), and BT16 (J) cell lines. Viability is shown as absorbance per well after fixation and staining with crystal violet. (K) Neurosphere volume after 7-d growth is significantly reduced by MRZ. Neurospheres were allowed to form, treated with 100 nM MRZ, and measured after 7 days. Statistics are generated by 1-way ANOVA. (L and M) BT16 neurosphere treated with Dimethyl sulfoxide (DMSO) control (L) versus 100 nM MRZ (M). Scale bar is 400 µm.

We applied CFZ and MRZ to 3 TYR-subgrouped cell lines (MAF-737A, BT12, and BT16) and 2 SHH-subgrouped lines (MAF-1298A and MAF-1337A) for 5 days to determine appropriate dosing and found that both compounds strongly inhibit growth at clinically achievable concentrations in all lines (CFZ average IC_50_ = 3.8 nM, MRZ average IC_50_ = 52 nM) (Figure 2A–E). There was no significant difference in CFZ dosing between the 2 subgroups, but the SHH subgrouped lines were noticeably less sensitive to MRZ, although this difference did not rise to the conventional definition of significance (*P* = .0855). Long-term growth assays conducted in a similar manner to a clonogenic assay (Figure 2F–J) found that inhibition of growth is maintained over time and that repeated smaller (sub 5-day IC50) doses are capable of substantially reducing growth potential. The MAF-737A, BT12, and BT16 lines are capable of growing in 3D culture as neurospheres, and a single treatment with MRZ significantly reduced the size of the resulting spheres over 7 days (Figure 2K and M).

MRZ induces strong proteasome inhibition and a rapid caspase activation, which is not essential for cell death, but may be variably inhibited by ROS scavenging

**Figure 3.**
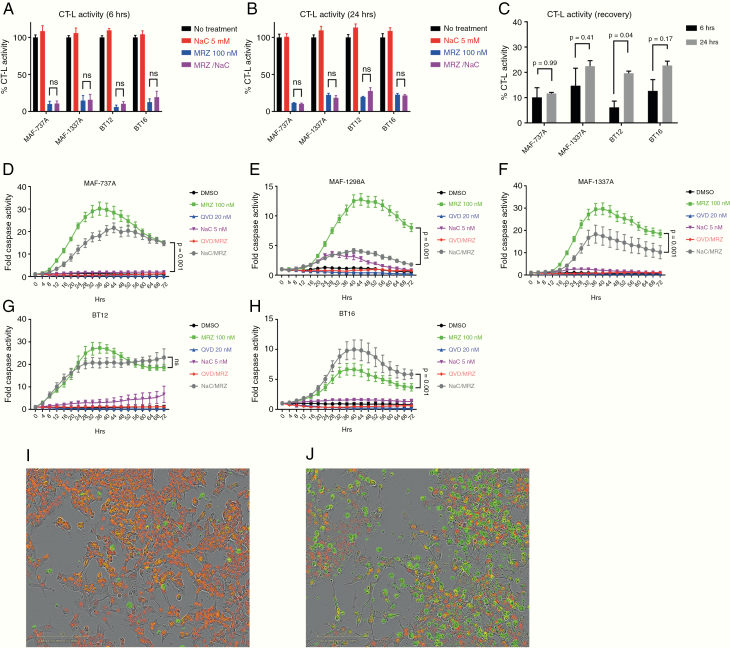
Marizomib (MRZ) induces strong proteasome inhibition and a rapid caspase activation, which is not essential for cell death, but may be variably inhibited by ROS scavenging. (A–C) Chymotrypsin-like activity of cells treated for either 6 or 24 h with 100 nM MRZ, 5 mM NaC, or both. (D–H) Caspase activity over 72 h by fluorescence microscopy in an Incucyte ZOOM for MAF-737A (D), MAF-1298A (E), MAF-1337A (F), BT12 (G), and BT16 (H). Statistics presented are from 2-way ANOVA. (I and J) BT16 cells treated for 24 h with either Dimethyl sulfoxide (DMSO) (I) or 100 nM MRZ (J). Cells are labeled with a nuclear red dye, and caspase activation is shown by green fluorescence. Scale bar is 200 µm. Statistics presented are for Dunnett’s multiple comparisons test.

In vitro application of 100 nM MRZ to cell lines results in rapid (6 hours) and moderately long-lasting (24 hours) inhibition of the proteasome, as measured by chymotrypsin-like activity (Figure 3A–C). It has been previously established that the reactive oxygen species scavenger NaC is capable of impairing the effects of several proteasome inhibitors,^15,24^ including MRZ,^18,20^ and rescuing cell proliferation. As it has been suggested that a potential mechanism of action by which NaC inhibits the effects of proteasome inhibitors is to directly bind to them,^24^ we conducted this experiment with or without the presence of NaC (Figure 3A and B). Proteasome activity is not affected by NaC, suggesting that direct binding and subsequent inactivation is not a relevant mechanism within the AT/RT context. MRZ also initiates the activation of caspases 3 and 7 beginning as soon as 6 hours post-treatment and clearly induced marked cell death by 24 hours (Figure 3D–J). Caspase activity generally peaks by 36 hours after treatment (Figure D–H) and gradually decreases. We attempted to rescue cell fate using either NaC or the pan-caspase inhibitor Q-VD-OPh (Figure 3C–G). Q-VD-OPh was capable of completely abolishing caspase activity at 20 µM. However, this impairment generally did not translate into significantly increased cell viability (Supplementary Figure S1), implying that while proteasome inhibition generates an apoptotic caspase response, caspase triggered apoptosis may not be the sole form of cell death induced. In the case of NaC, 5 mM strongly reduced caspase signaling in the MAF-737A, MAF-1298A, and MAF-1337A lines, but had either no effect (BT12) or increased caspase activity (BT16) in the other 2 lines. Curiously, in spite of the significant NaC-associated increase in caspase activity detected in the BT16 line, when cotreated with MRZ, there was no decrease in cell viability (Supplementary Figure S1). As noted, NaC was somewhat toxic by itself in BT16 cells (Supplementary Figure S1). Additionally, while NaC reduced caspase signaling in 3 lines, this did not always result in a rescue of cell viability. This is supported by measurements of cell confluence (Supplementary Figure S2) showing increased cell confluence in MAF-1298A cells, but no increase in MAF-737A or MAF-1337A cells.

### Proteasome Inhibition Is Not Synergistic With Autophagy Inhibition or Radiation Therapy, but Induces Oxidative Stress

**Figure 4.**
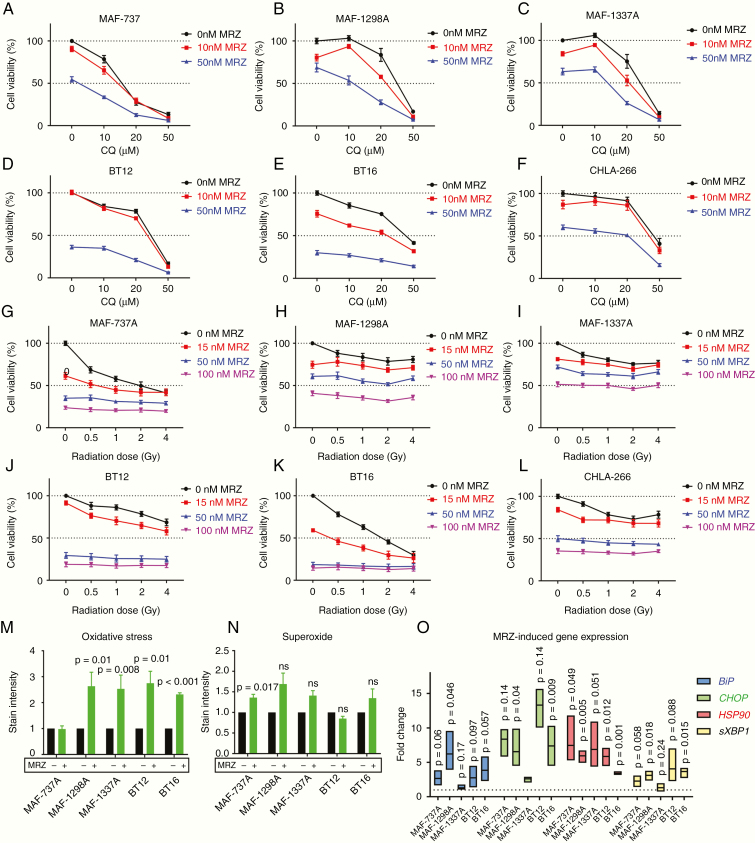
Proteasome inhibition is generally not synergistic with either autophagy inhibition or radiation therapy, but induces oxidative stress. (A–F) End point measures of viability by CellTiter Glo, shown as % of Dimethyl sulfoxide (DMSO) control for MAF-737A (A), MAF-1298A (B), MAF-1337A (C), BT12 (D), BT16 (E), and CHLA-266 (F) cell lines treated with varying concentrations of marizomib (MRZ) and chloroquine (CQ). (G–L) End point measures of viability by CellTiter Glo, shown as % of DMSO control for MAF-737A (G), MAF-1298A (H), MAF-1337A (I), BT12 (J), BT16 (K), and CHLA-266 (L) cell lines treated with varying concentrations of MRZ and Cs-137 radiation. Statistics shown for (A–L) are for 2-way ANOVA. (M–N) Cells treated for 24 h with 100 nM MRZ and stained for ROS (M) and superoxide (N) and quantified by flow cytometry. Statistics shown are Dunnett’s multiple comparisons test of MRZ-treated versus DMSO control for normalized data. (O) Real-time quantitative PCR for *HSPA5* (*BiP*), *CHOP*, *HSP90*, and spliced *XBP1* (*sXBP1*) after 24-h treatment with 100 nM MRZ. Statistics shown are 1-tailed Student’s *T*-test compared with DMSO-treated controls, *n* = 3.

It has been reported that autophagy inhibition synergizes with proteasome inhibition in to kill tumor cells in extra-CNS myeloid/rhabdoid tumors,^27^ and this combination is rational and has seen positive results in other contexts.^28^ Conversely, our group has previously reported in BT12 and BT16 cells that there was no synergy between genetic or pharmacologic inhibition of autophagy in standard chemotherapy.^29^ Radiation is also a common first-line therapy in AT/RT in combination with HDC. Accordingly, we combined varying levels of MRZ with either the late-stage autophagy inhibitor chloroquine (CQ) (Figure 4A–E) or radiation (Figure 4G–L). However, MRZ and CQ combination did not result in robust synergy. The 2 SHH-grouped cell lines (MAF-11298A and MAF-1337A) did show some degree of mild synergy with specific doses of CQ, but Bliss analysis shows that this occurs only within specific dose bands for each compound and is not a general property (Supplementary Table S2). Combination with radiation (Figure 4G–L) demonstrated that MRZ does not sensitize these cells to radiation and may actually have a protective effect. We fluorescently stained the cells for both oxidative stress and superoxide after 24 hours of treatment with 100 nM MRZ, and quantified the intensity of the staining by flow cytometry. From this, we found that MRZ significantly increased oxidative stress in all cell lines save MAF-737A, which was the only line to show a significant increase in superoxide (Figure M and N). Supporting this was our observation that superoxide dismutase (*SOD1*) was generally not significantly upregulated following MRZ treatment (Supplementary Figure S3).We examined the expression of several genes associated with the unfolded protein response (Figure 4O), and found that *HSPA5* (*BiP*), *CHOP*, *HSP90*, and spliced *XBP1* (*sXBP1*) were significantly upregulated in most cell lines, revealing a potential mechanism for apoptosis in increased proteostatic stress.^30^

### MRZ Suppresses AT/RT Tumor Growth In Vivo

**Figure 5.**
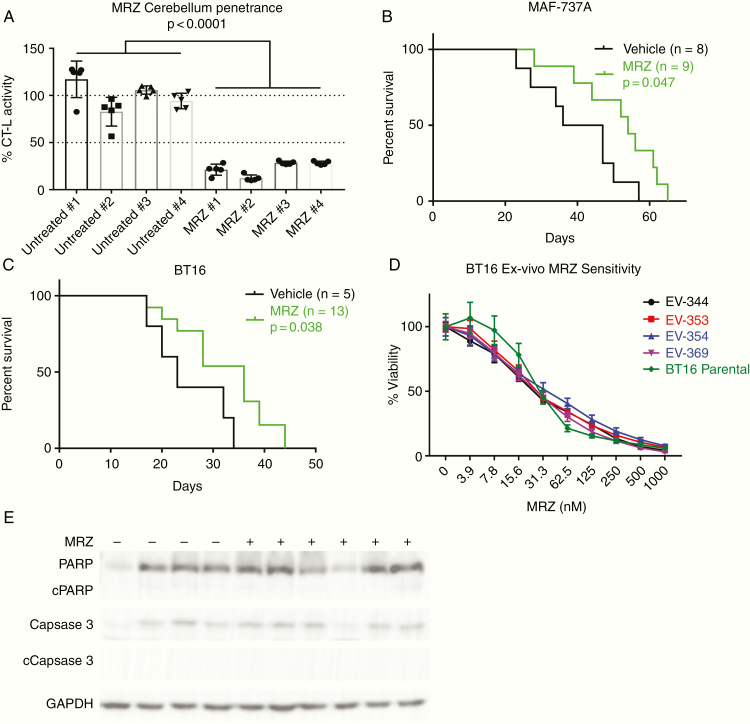
Marizomib (MRZ) suppresses atypical teratoid/thabdoid tumor (AT/RT) tumor growth in vivo. (A) Chymotrypsin-like activity of disaggregated single cells isolated from mouse cerebellums 1 h post-injection with either 200 µg/kg MRZ or an equivalent volume of vehicle. Statistics shown are for 1-way ANOVA, *n* = 8. (B, C) Kaplan–Meier graphs showing cerebellar xenografts of MAF-737A (B) or BT16 (C) cells, treated twice weekly with either 200 µg/kg (B) or 150 µg/kg (C) MRZ. (D) Dose–response curves to 5-day treatment from BT16 cells isolated from mice in the treatment group of (C). (E) Western blot analysis of tumors extracted from mice from (B), blotting for total and cleaved PARP, total and cleaved Caspase 3, and GAPDH loading control.

MRZ is capable of penetrating past the BBB into the cerebellum, as shown by a decrease in CT-L activity (Figure 5A), and is capable of significantly extending the median lifespan of mice bearing intracranial BT16 and MAF-737A intracranial tumors (Figure 5B and C) by an average of 12.75 days (43% greater than median control survival). The doses of 150 and 200 µg/kg were well-tolerated, and BT16 cells isolated ex vivo after animal sacrifice did not display any enhanced resistance to MRZ (Figure 5D). BT16 xenograft median survival was extended from 23 to 36 days (*P* = .0384), and MAF-737A median survival was extended from 41.5 to 54 days (*P* = .0474). Western blot analysis of tumors collected immediately post-sacrifice did not show significant differences in cleaved PARP or cleaved caspase 3 (Figure 5E), and there was also no noticeable difference in buildup of ubiquitinated proteins (Supplementary Figure S4).

## Discussion

Targeting the proteasome in RT was considered as a potential therapy option a number of years ago. Unfortunately, at that time, BTZ was the only such inhibitor available. A study by the Pediatric Preclinical Testing Program^31^ evaluated its use in a variety of tumor subtypes including AT/RT cell lines and found the drug to be uniformly active with low IC_50_ levels. In vivo, BTZ treatment resulted in prolonged Event-free survival in glioblastoma, medulloblastoma, and rhabdomyosarcoma panels, but overall objective responses were restricted to ALL xenografts. With the development of MRZ, improved drug effectiveness and penetration of the BBB allows for reevaluation of proteasome inhibition in brain tumor populations. We have demonstrated here that MRZ is capable of rapidly killing AT/RT cells and that it can cross the BBB and inhibit tumor growth in a murine model.

The precise mechanism of rhabdoid cell killing by MRZ remains to be uncovered, for although we have shown that ROS levels and general oxidative stress are elevated upon MRZ treatment, this effect is neither consistent nor consistently alleviated by antioxidant supplementation. Our rescue effects using Q-VD-OPh and NaC are less dramatic than those reported in glioblastoma models.^20^ We also did not observe a previously reported effect of NaC directly binding and inhibiting proteasome inhibitors,^24^ although MRZ was not among the inhibitors tested in that study. The AT/RT context appears to further differentiate from other tumor models, in that we did not find the expected synergy between MRZ and autophagy inhibitors. This is consistent with our previous work suggesting AT/RT is not autophagy dependent.^29^ Radiation therapy also proved to be a poor combination, although this is perhaps not unexpected, given that MRZ may work by enhancing the already-high level of Endoplasmic reticulum stress that AT/RT cells suffer from, and this stress could be attenuated by retarding the cell cycle via radiation-induced DNA damage.^32^

MRZ has been shown to improve animal survival in models of both glioblastoma^20,33^ and diffuse midline glioma,^34^ and a comparable improvement is seen in our AT/RT model using identical dosing schedules. However, in spite of this survival advantage, the level of proteasome inhibition seen in the cerebellum is much less intense than what can be achieved in vitro, and examination of the tumors post-treatment showed little to no evidence of apoptosis. Western blotting for both total ubiquitin and K48-linked ubiquitin (Supplementary Figure S4) showed no accumulation in the treated tumors, as would be expected from a proteasome inhibitor (although this is likely due to time elapsed since last dose, as sacrifice time was independent of the last dose administration). MRZ is capable of entering the brain, but the extremely short half-life^33^ suggests that better results may be obtained via either more frequent dosing, or administration by a technique such as convection-enhanced delivery. Any method of application would require a phase I trial to establish dosing, as marizomib has not been used in the pediatric context.

Proteasome inhibition has already demonstrated great potential in adult glioma, both as a single agent and in combination with other chemotherapies.^35^ We have shown here that MRZ is also efficacious in pediatric AT/RT tumor models as a single agent, prolonging survival even at concentrations too low to induce significant cell death.

## Supplementary Material

vdaa051_suppl_supplementary_Figure_S1

vdaa051_suppl_supplementary_Figure_S2

vdaa051_suppl_supplementary_Figure_S3

vdaa051_suppl_supplementary_Figure_S4

vdaa051_suppl_supplementary_Figure_S5

vdaa051_suppl_supplementary_Table_S1

vdaa051_suppl_supplementary_Table_S2

vdaa051_suppl_Supplementary_Table_and_Figure_Legends
