# Supplementary figures and images for: Proteasome inhibition as a therapeutic approach in atypical teratoid/rhabdoid tumors
Source: Neurooncol Adv. 2020 Apr 14;2(1):vdaa051. doi: 10.1093/noajnl/vdaa051 (PMC7236404; doi:10.1093/noajnl/vdaa051)

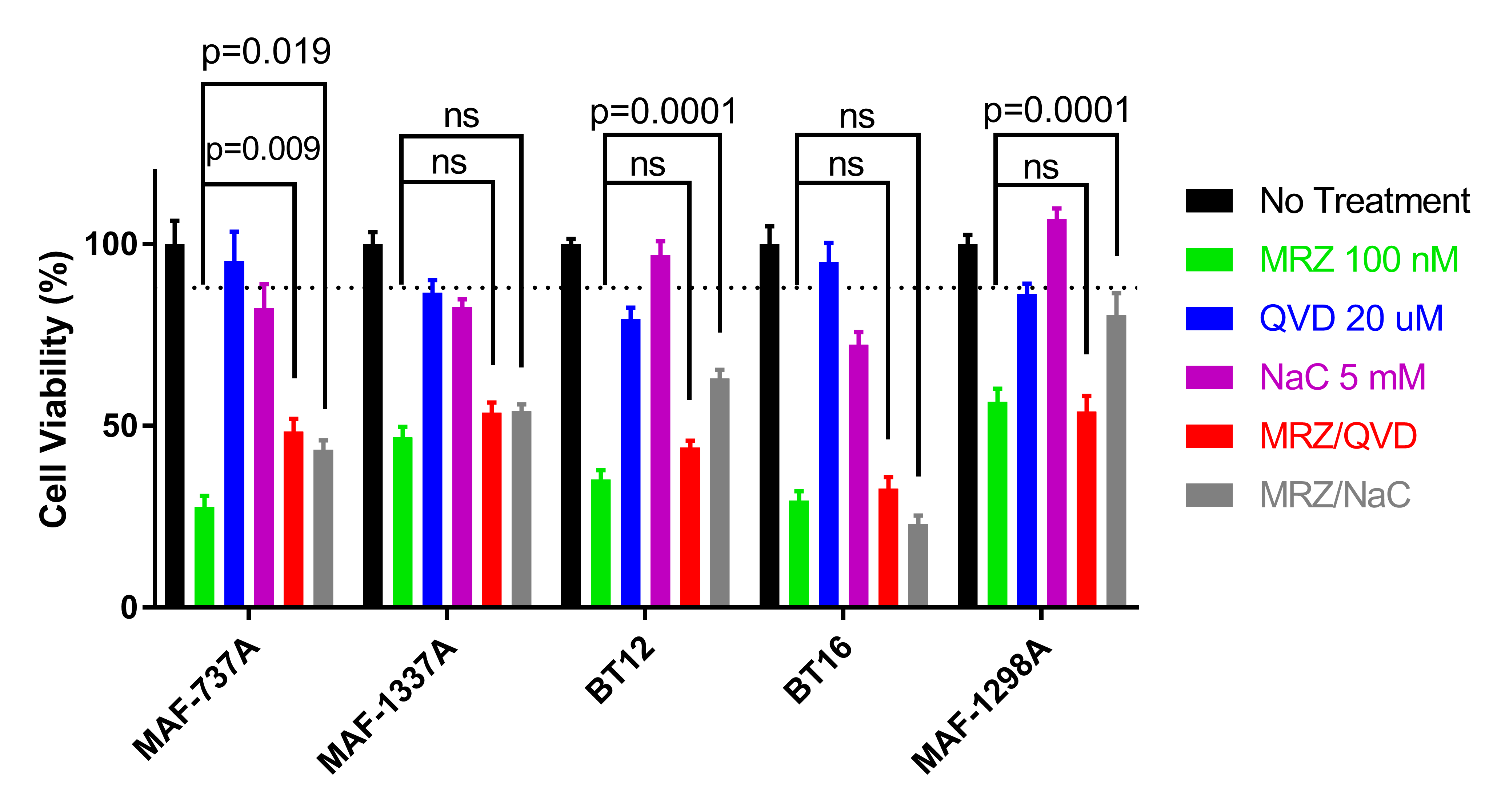

Supplement: vdaa051_suppl_supplementary_Figure_S1 [file vdaa051_suppl_supplementary_figure_s1.png]

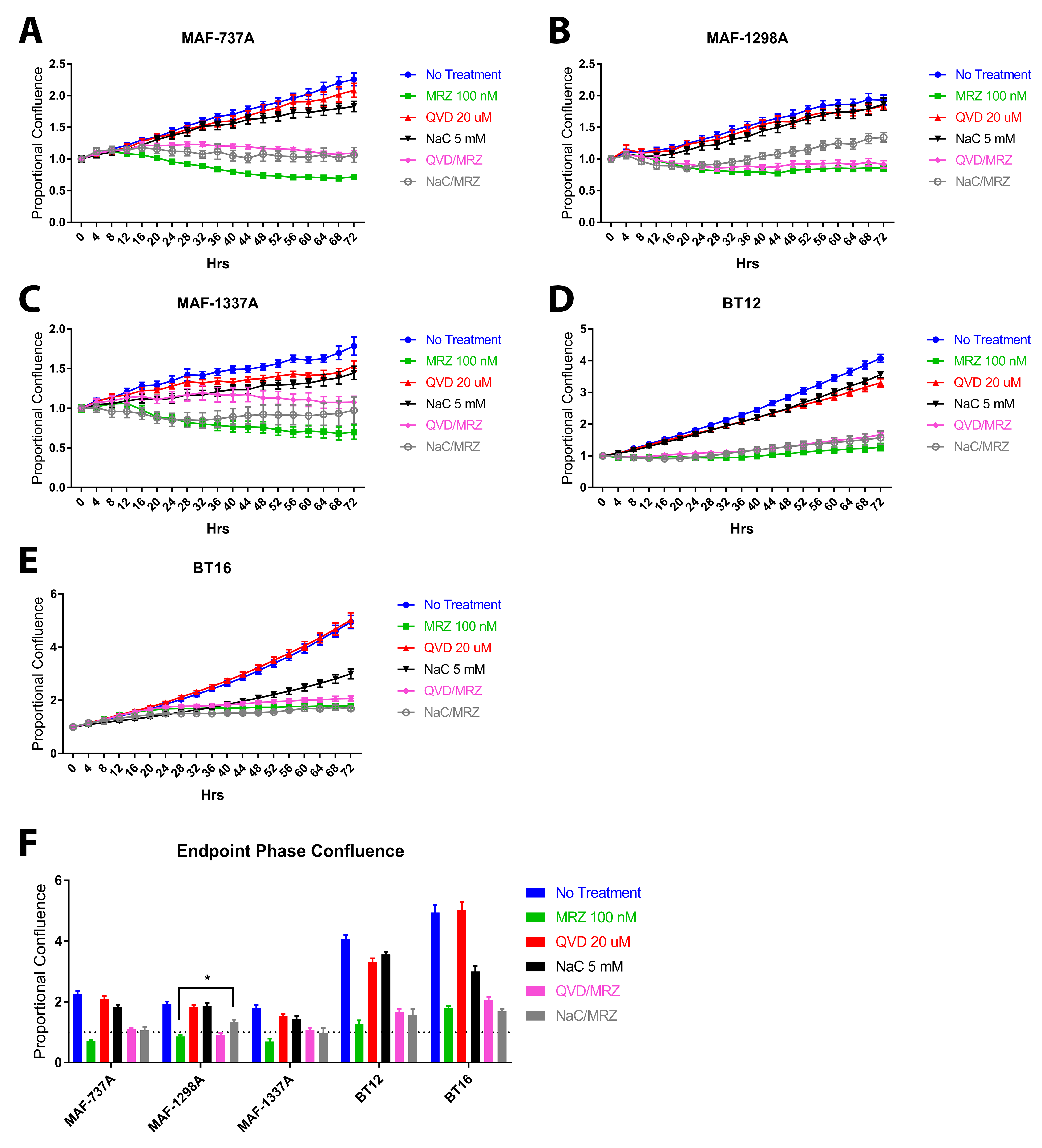

Supplement: vdaa051_suppl_supplementary_Figure_S2 [file vdaa051_suppl_supplementary_figure_s2.png]

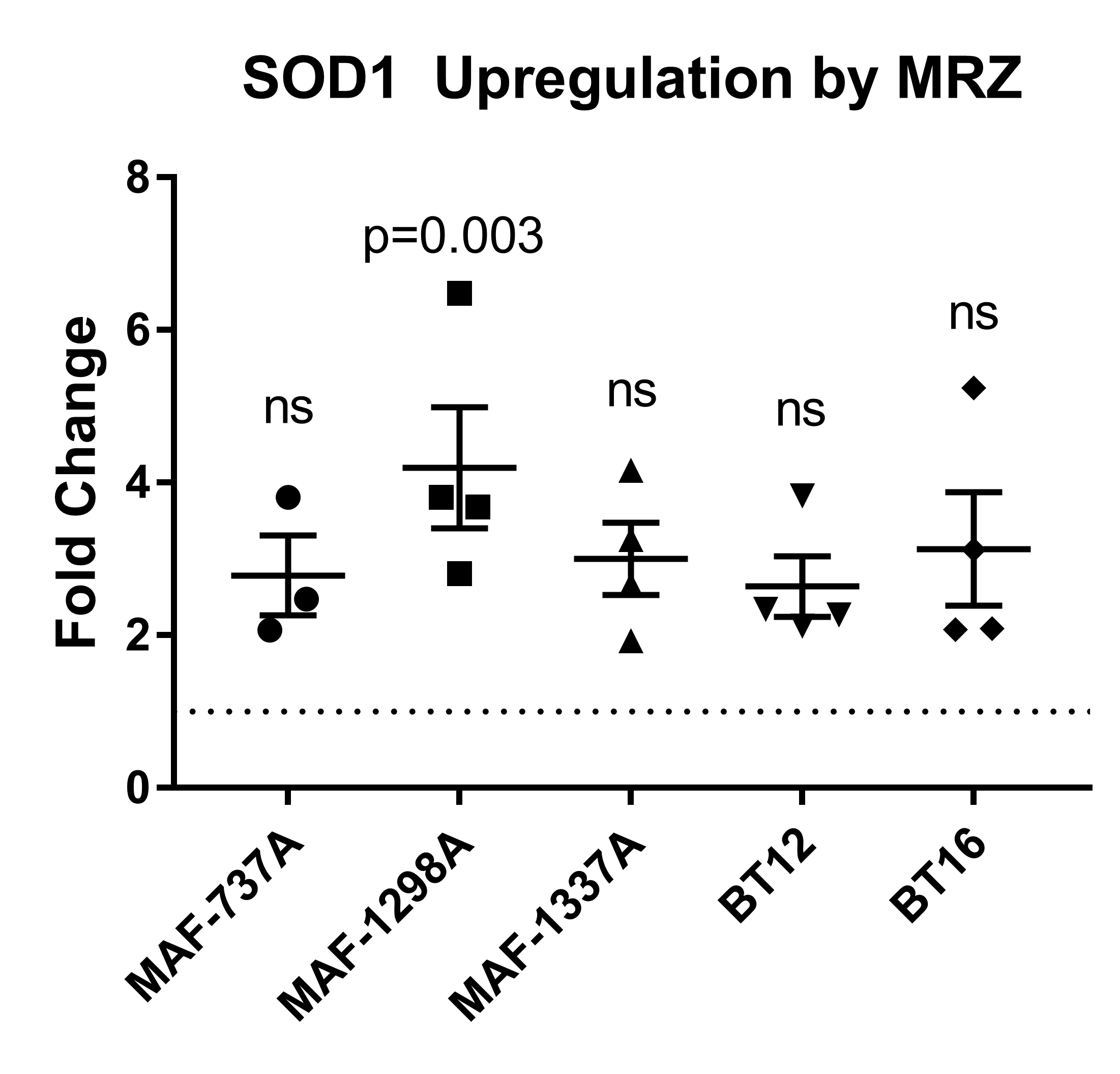

Supplement: vdaa051_suppl_supplementary_Figure_S3 [file vdaa051_suppl_supplementary_figure_s3.png]

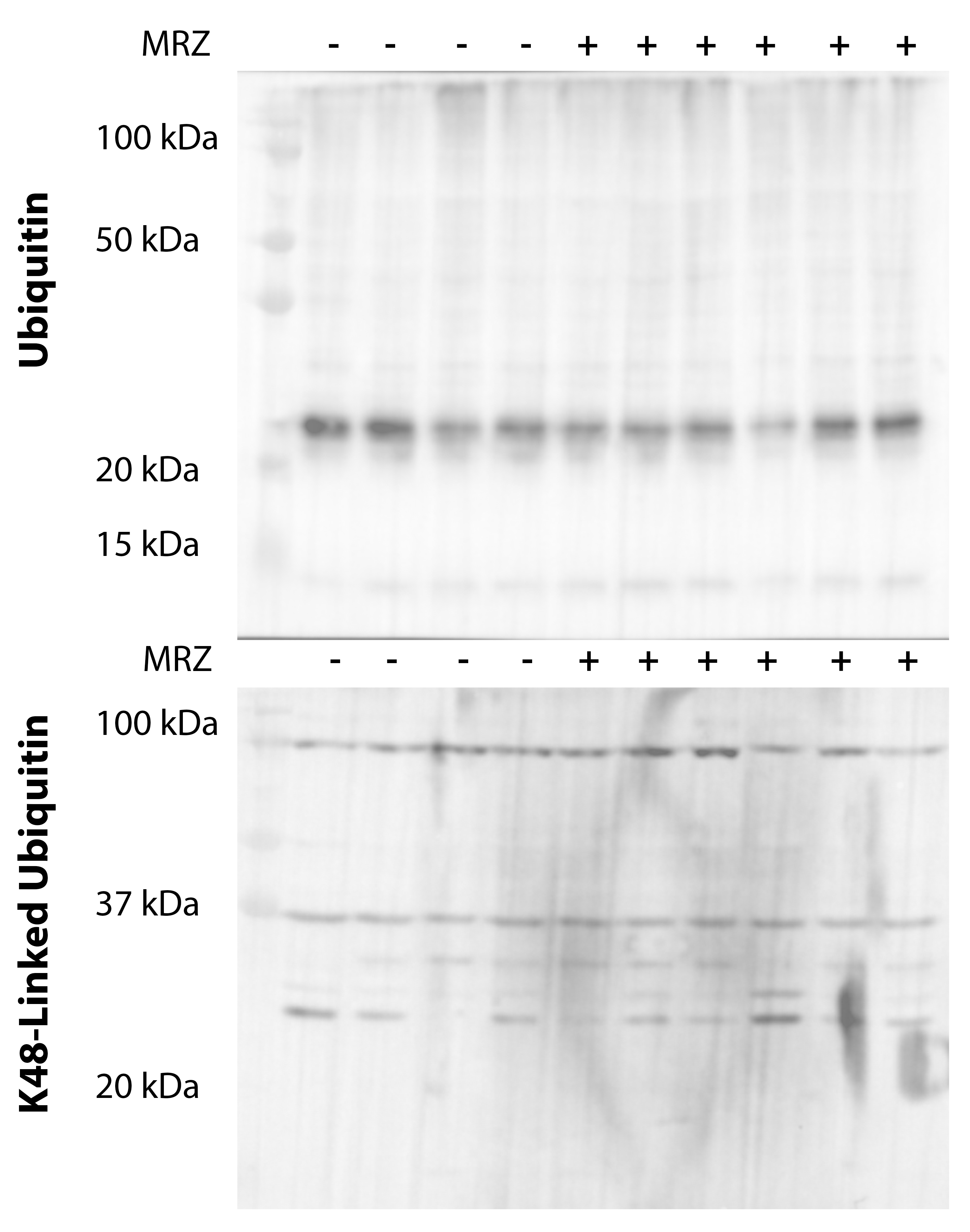

Supplement: vdaa051_suppl_supplementary_Figure_S4 [file vdaa051_suppl_supplementary_figure_s4.png]

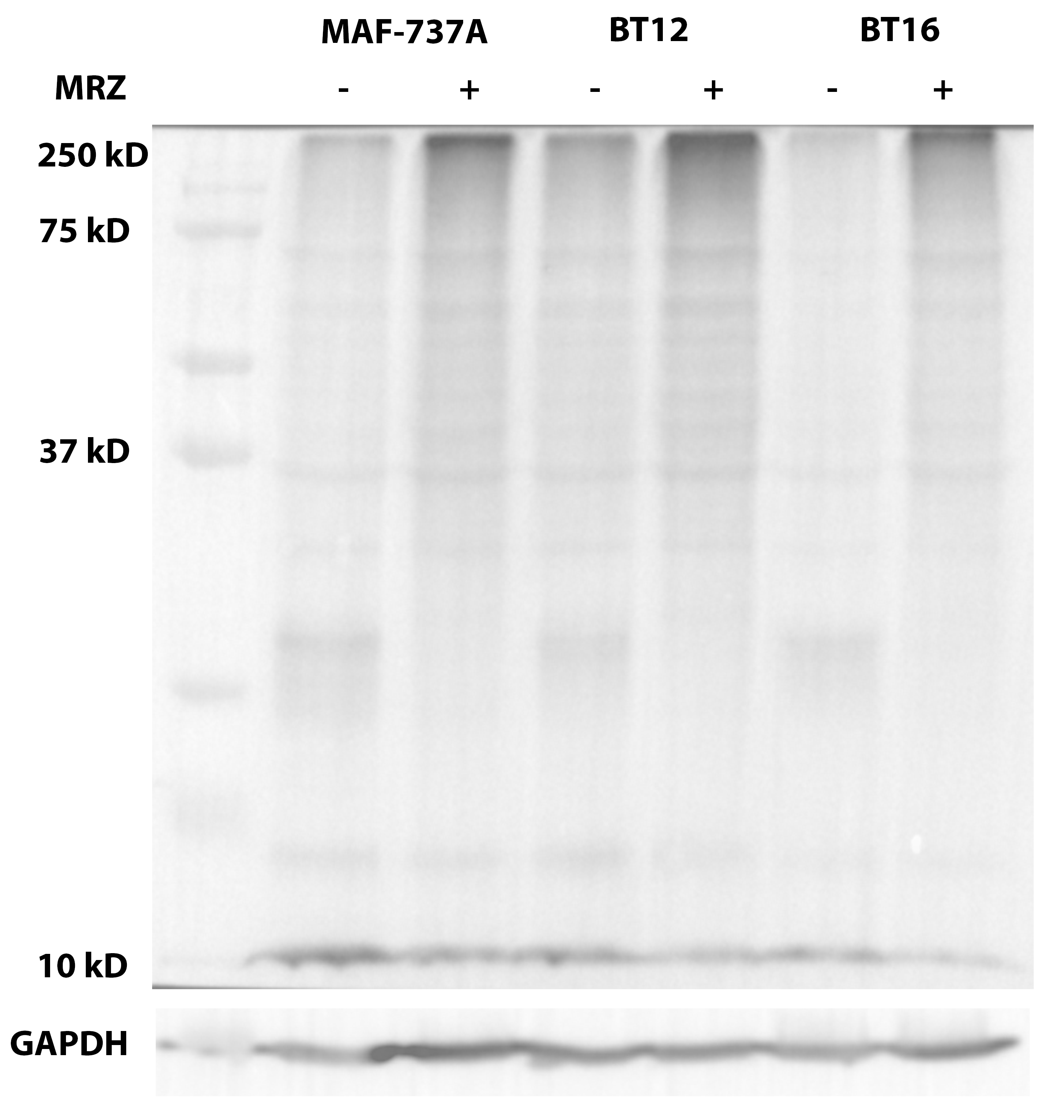

Supplement: vdaa051_suppl_supplementary_Figure_S5 [file vdaa051_suppl_supplementary_figure_s5.png]
