# Supplementary material for: Proteasome inhibition as a therapeutic approach in atypical teratoid/rhabdoid tumors
Source: Neurooncol Adv. 2020 Apr 14;2(1):vdaa051. doi: 10.1093/noajnl/vdaa051 (PMC7236404; doi:10.1093/noajnl/vdaa051)
Supplement: vdaa051_suppl_supplementary_Table_S1 [file vdaa051_suppl_supplementary_table_s1.docx]

| **Table S1: NCI AOD Results** | **MAF-737A** | | **BT12** | | **BT16** | |
| --- | --- | --- | --- | --- | --- | --- |
| **Compound** | **% Viability** | **Std Dev** | **% Viability** | **Std Dev** | **% Viability** | **Std Dev** |
| Abiraterone | 89.85 | 11.56 | 68.22 | 30.75 | 72.60 | 30.09 |
| Afatinib | 92.68 | 5.71 | 89.33 | 7.35 | 16.07 | 1.92 |
| Alectinib | 101.89 | 2.35 | 97.80 | 3.21 | 100.30 | 16.20 |
| Allopurinol | 96.53 | 15.67 | 88.78 | 5.75 | 91.55 | 1.99 |
| Altretamine | 109.51 | 4.31 | 103.70 | 11.90 | 99.41 | 3.20 |
| Amifostine | 100.26 | 14.67 | 93.44 | 4.28 | 90.23 | 2.64 |
| Aminolevulinic acid hydrochloride | 121.18 | 12.76 | 118.23 | 9.15 | 96.72 | 7.88 |
| Anastrozole | 111.80 | 9.72 | 108.74 | 17.17 | 98.52 | 7.13 |
| Arsenic trioxide | 104.86 | 6.23 | 99.67 | 0.66 | 100.07 | 1.02 |
| Axitinib | 96.55 | 5.60 | 92.76 | 7.44 | 94.33 | 22.84 |
| Azacitidine | 105.47 | 4.29 | 91.95 | 9.31 | 82.36 | 9.11 |
| Belinostat | 113.06 | 2.80 | 98.88 | 18.91 | 54.06 | 13.67 |
| Bendamustine hydrochloride | 94.78 | 6.86 | 94.94 | 2.47 | 91.68 | 10.28 |
| Bleomycin sulfate | 63.45 | 8.82 | 57.52 | 19.21 | 25.83 | 8.03 |
| Bortezomib | 35.08 | 29.47 | 4.00 | 0.74 | 7.08 | 3.04 |
| Bosutinib | 86.00 | 10.55 | 78.27 | 2.12 | 52.61 | 6.37 |
| Busulfan | 108.32 | 7.97 | 98.02 | 6.02 | 97.35 | 12.86 |
| Cabazitaxel | 46.70 | 3.95 | 5.09 | 1.73 | 10.57 | 4.52 |
| Cabozantinib | 90.86 | 5.22 | 94.46 | 1.77 | 110.45 | 20.42 |
| Capecitabine | 110.99 | 8.37 | 107.80 | 16.38 | 91.44 | 6.99 |
| Carboplatin | 110.66 | 7.12 | 96.28 | 14.47 | 87.40 | 18.02 |
| Carfilzomib | 6.37 | 1.89 | 2.31 | 0.97 | 1.91 | 0.62 |
| Carmustine | 101.89 | 12.82 | 93.72 | 6.89 | 98.71 | 14.84 |
| Celecoxib | 112.34 | 9.37 | 100.37 | 19.95 | 101.59 | 20.02 |
| Ceritinib | 92.10 | 5.05 | 93.02 | 4.01 | 101.88 | 6.05 |
| Chlorambucil | 97.10 | 12.07 | 88.59 | 3.60 | 89.66 | 9.45 |
| Cisplatin | 111.67 | 4.64 | 107.80 | 9.94 | 88.55 | 10.57 |
| Cladribine | 94.76 | 3.59 | 100.67 | 5.19 | 86.73 | 9.39 |
| Clofarabine | 100.10 | 7.54 | 103.40 | 12.49 | 88.66 | 9.25 |
| Cobimetinib | 74.35 | 2.57 | 54.84 | 4.14 | 56.10 | 4.68 |
| Crizotinib | 100.00 | 4.22 | 97.18 | 4.60 | 95.65 | 3.74 |
| Cyclophosphamide | 111.98 | 5.74 | 101.99 | 12.90 | 96.29 | 8.92 |
| Cytarabine hydrochloride | 85.78 | 2.36 | 80.33 | 11.68 | 26.79 | 5.86 |
| Dabrafenib mesylate | 98.93 | 18.16 | 74.72 | 11.74 | 105.48 | 13.45 |
| Dacarbazine | 90.50 | 3.42 | 90.53 | 8.49 | 98.14 | 7.86 |
| Dactinomycin | 12.50 | 3.41 | 3.24 | 1.45 | 4.44 | 1.48 |
| Dasatinib | 29.77 | 4.53 | 58.44 | 3.64 | 27.24 | 3.40 |
| Daunorubicin hydrochloride | 49.09 | 1.96 | 17.61 | 11.08 | 13.92 | 3.76 |
|  | **MAF-737A** | | **BT12** | | **BT16** | |
| **Compound** | **% Viability** | **Std Dev** | **% Viability** | **Std Dev** | **% Viability** | **Std Dev** |
| Decitabine | 93.65 | 4.12 | 102.65 | 9.22 | 72.06 | 5.83 |
| Dexrazoxane | 112.03 | 6.22 | 103.97 | 19.39 | 96.06 | 12.11 |
| Docetaxel | 47.63 | 6.33 | 6.20 | 1.61 | 8.97 | 1.86 |
| Doxorubicin hydrochloride | 53.12 | 10.87 | 20.20 | 8.77 | 19.16 | 2.42 |
| Enzalutamide | 92.13 | 7.14 | 79.42 | 14.29 | 99.58 | 10.64 |
| Epirubicin hydrochloride | 57.76 | 9.26 | 31.13 | 12.19 | 22.25 | 6.84 |
| Erismodegib | 89.40 | 7.45 | 86.29 | 1.50 | 104.54 | 12.15 |
| Erlotinib hydrochloride | 106.86 | 4.36 | 105.10 | 29.17 | 76.24 | 19.56 |
| Estramustine phosphate sodium | 95.63 | 7.88 | 101.30 | 2.88 | 107.85 | 2.94 |
| Etoposide | 77.31 | 14.19 | 55.36 | 17.80 | 60.58 | 7.81 |
| Everolimus | 52.16 | 9.95 | 45.91 | 2.27 | 30.48 | 6.10 |
| Exemestane | 103.92 | 11.82 | 100.68 | 12.77 | 91.55 | 16.69 |
| Floxuridine | 109.31 | 2.57 | 80.90 | 11.26 | 62.41 | 7.93 |
| Fludarabine phosphate | 111.43 | 4.48 | 100.59 | 9.93 | 81.05 | 8.33 |
| Fluorouracil | 100.72 | 2.73 | 94.13 | 6.85 | 101.46 | 7.26 |
| Fulvestrant | 110.15 | 8.45 | 97.26 | 6.68 | 106.92 | 13.47 |
| Gefitinib | 100.75 | 2.51 | 99.17 | 9.09 | 63.62 | 9.53 |
| Gemcitabine hydrochloride | 55.95 | 2.97 | 8.48 | 3.83 | 7.97 | 3.47 |
| Hydroxyurea | 102.48 | 8.13 | 99.04 | 2.09 | 89.98 | 7.69 |
| Ibrutinib | 98.66 | 3.92 | 105.69 | 14.15 | 17.80 | 2.54 |
| Idarubicin hydrochloride | 44.74 | 5.31 | 20.46 | 10.11 | 8.34 | 0.75 |
| Idelalisib | 99.12 | 5.24 | 103.36 | 17.64 | 84.21 | 8.11 |
| Ifosfamide | 115.20 | 11.13 | 110.93 | 11.83 | 89.80 | 15.59 |
| Imatinib | 89.16 | 2.28 | 76.40 | 2.59 | 82.48 | 12.66 |
| Imiquimod | 86.21 | 25.94 | 98.82 | 7.49 | 98.43 | 20.41 |
| Irinotecan hydrochloride | 92.53 | 14.16 | 77.24 | 1.48 | 85.31 | 4.41 |
| Ixabepilone | 51.58 | 4.17 | 5.38 | 1.15 | 8.73 | 0.18 |
| Ixazomib citrate | 98.74 | 9.41 | 67.53 | 43.07 | 78.26 | 40.78 |
| Lapatinib | 95.84 | 9.15 | 95.57 | 4.63 | 84.69 | 27.33 |
| Lenalidomide | 110.87 | 9.32 | 108.01 | 8.81 | 98.37 | 7.68 |
| Lenvatinib | 95.61 | 5.21 | 82.78 | 4.94 | 80.98 | 8.54 |
| Letrozole | 109.85 | 5.40 | 104.07 | 10.92 | 88.76 | 8.54 |
| Lomustine | 112.75 | 3.63 | 117.88 | 7.74 | 121.57 | 9.75 |
| Mechlorethamine hydrochloride | 98.62 | 1.98 | 100.68 | 4.13 | 86.58 | 13.65 |
| Megestrol acetate | 98.72 | 7.50 | 93.64 | 6.75 | 93.75 | 5.47 |
| Melphalan hydrochloride | 102.60 | 1.79 | 88.59 | 3.08 | 95.50 | 8.88 |
| Mercaptopurine | 106.79 | 5.71 | 101.41 | 4.79 | 91.54 | 8.35 |
| Methotrexate | 117.00 | 5.09 | 60.99 | 14.29 | 59.93 | 16.95 |
|  | **MAF-737A** | | **BT12** | | **BT16** | |
| **Compound** | **% Viability** | **Std Dev** | **% Viability** | **Std Dev** | **% Viability** | **Std Dev** |
| Methoxsalen | 106.85 | 1.02 | 114.73 | 5.99 | 103.62 | 10.58 |
| Mitomycin | 75.98 | 8.30 | 22.98 | 7.61 | 16.83 | 3.20 |
| Mitotane | 102.14 | 9.65 | 105.55 | 16.48 | 91.17 | 10.07 |
| Mitoxantrone | 51.63 | 7.43 | 29.03 | 8.05 | 12.58 | 1.94 |
| Nelarabine | 108.60 | 1.95 | 109.44 | 22.75 | 103.53 | 12.04 |
| Nilotinib | 102.45 | 6.63 | 100.50 | 2.81 | 92.69 | 22.90 |
| Niraparib hydrochloride | 81.41 | 3.73 | 84.50 | 3.89 | 80.85 | 5.12 |
| No Treatment | 100.00 | 0.00 | 100.00 | 0.00 | 100.00 | 0.00 |
| Olaparib | 111.26 | 6.31 | 106.87 | 12.06 | 97.51 | 6.29 |
| Omacetaxine mepesuccinate | 28.85 | 6.84 | 9.64 | 7.52 | 4.85 | 2.97 |
| Osimertinib | 85.62 | 6.38 | 88.46 | 1.68 | 44.33 | 6.02 |
| Oxaliplatin | 106.65 | 6.28 | 92.12 | 22.16 | 89.17 | 13.79 |
| Paclitaxel | 52.20 | 8.54 | 7.41 | 3.61 | 10.73 | 4.20 |
| Palbociclib | 90.80 | 14.22 | 90.33 | 8.38 | 92.26 | 12.39 |
| Panobinostat | 29.69 | 13.02 | 13.69 | 7.69 | 13.43 | 0.12 |
| Pazopanib hydrochloride | 88.92 | 13.70 | 94.74 | 2.63 | 105.76 | 8.54 |
| Pemetrexed | 113.55 | 0.32 | 95.88 | 21.65 | 101.47 | 11.48 |
| Pentostatin | 110.86 | 2.87 | 118.94 | 14.88 | 109.80 | 7.85 |
| Pipobroman | 107.62 | 5.49 | 106.89 | 13.49 | 104.42 | 11.14 |
| Plerixafor | 110.14 | 32.42 | 96.34 | 11.46 | 100.30 | 15.42 |
| Plicamycin | 15.97 | 2.99 | 0.46 | 0.39 | 2.39 | 2.00 |
| Pomalidomide | 128.96 | 7.37 | 110.73 | 9.23 | 106.03 | 0.89 |
| Ponatinib | 79.72 | 7.94 | 70.33 | 9.49 | 93.21 | 13.10 |
| Pralatrexate | 113.17 | 7.60 | 51.05 | 17.70 | 51.77 | 17.48 |
| Procarbazine hydrochloride | 106.33 | 12.85 | 97.29 | 8.95 | 90.70 | 18.18 |
| Raloxifene | 105.28 | 10.80 | 100.35 | 15.60 | 95.97 | 5.25 |
| Regorafenib | 101.53 | 23.64 | 97.68 | 3.56 | 113.05 | 18.19 |
| Ribociclib | 100.55 | 4.83 | 75.53 | 17.50 | 94.98 | 7.93 |
| Romidepsin | 5.40 | 2.38 | 1.32 | 1.50 | 3.80 | 2.38 |
| Rucaparib phosphate | 99.90 | 15.96 | 95.64 | 13.89 | 88.37 | 7.97 |
| Sirolimus | 45.95 | 4.65 | 44.89 | 1.82 | 28.67 | 2.49 |
| Sorafenib | 121.87 | 7.32 | 110.26 | 13.06 | 98.02 | 12.64 |
| Streptozocin | 111.30 | 3.50 | 99.97 | 2.80 | 92.74 | 5.19 |
| Sunitinib | 107.53 | 4.65 | 102.30 | 5.76 | 114.46 | 4.90 |
| Tamoxifen citrate | 96.93 | 6.13 | 92.80 | 0.23 | 99.90 | 10.55 |
| Temozolomide | 103.31 | 9.28 | 100.14 | 5.36 | 101.11 | 4.81 |
| Temsirolimus | 50.81 | 5.22 | 46.31 | 6.01 | 30.80 | 3.75 |
| Teniposide | 66.88 | 7.08 | 40.26 | 13.72 | 20.06 | 8.12 |
|  | **MAF-737A** | | **BT12** | | **BT16** | |
| **Compound** | **% Viability** | **Std Dev** | **% Viability** | **Std Dev** | **% Viability** | **Std Dev** |
| Thalidomide | 106.42 | 2.26 | 110.64 | 10.56 | 103.65 | 4.52 |
| Thioguanine | 103.32 | 15.17 | 98.44 | 4.61 | 92.72 | 3.25 |
| Thiotepa | 101.60 | 3.22 | 97.13 | 14.73 | 92.89 | 6.69 |
| Topotecan hydrochloride | 49.75 | 5.78 | 15.40 | 4.78 | 8.64 | 1.86 |
| Trametinib | 69.78 | 0.67 | 32.75 | 5.95 | 25.40 | 5.21 |
| Tretinoin | 113.32 | 4.70 | 107.69 | 10.76 | 72.90 | 12.87 |
| Triethylenemelamine | 74.49 | 8.78 | 82.11 | 14.99 | 69.52 | 1.88 |
| Trifluridine | 110.97 | 5.56 | 105.90 | 11.65 | 107.13 | 6.03 |
| Uracil mustard | 108.42 | 17.82 | 104.87 | 13.79 | 92.18 | 13.31 |
| Uridine triacetate | 103.67 | 3.04 | 113.00 | 20.37 | 90.78 | 13.21 |
| Valrubicin | 72.00 | 11.98 | 62.18 | 20.09 | 38.20 | 16.53 |
| Vandetanib | 80.75 | 6.07 | 71.99 | 10.20 | 95.99 | 12.22 |
| Vemurafenib | 101.58 | 14.50 | 94.31 | 1.48 | 109.52 | 18.73 |
| Venetoclax | 98.06 | 11.87 | 92.73 | 10.10 | 96.06 | 13.78 |
| Vinblastine sulfate | 39.12 | 3.63 | 4.04 | 2.76 | 8.17 | 1.93 |
| Vincristine sulfate | 43.60 | 3.27 | 5.16 | 3.15 | 12.88 | 3.99 |
| Vinorelbine tartrate | 64.44 | 15.81 | 22.83 | 14.65 | 57.02 | 11.52 |
| Vismodegib | 105.87 | 8.69 | 101.82 | 5.43 | 89.68 | 9.16 |
| Vorinostat | 108.54 | 6.91 | 96.46 | 1.78 | 77.14 | 3.52 |
| Zoledronic acid | 98.16 | 11.42 | 94.35 | 6.46 | 105.34 | 7.93 |
