# Supplementary material for: Proteasome inhibition as a therapeutic approach in atypical teratoid/rhabdoid tumors
Source: Neurooncol Adv. 2020 Apr 14;2(1):vdaa051. doi: 10.1093/noajnl/vdaa051 (PMC7236404; doi:10.1093/noajnl/vdaa051)
Supplement: vdaa051_suppl_supplementary_Table_S2 [file vdaa051_suppl_supplementary_table_s2.docx]

| Table S2: Bliss Synergy analysis of MRZ and CQ combination treatments | | | | | | |
| --- | --- | --- | --- | --- | --- | --- |
| **Bliss Synergy Score** | |  |  |  |  |  |
| **MAF-1298A** | | 10 | 20 | 50 | 75 | CQ (uM) |
|  | 10 | 1.03 | 0.87 | 0.92 | 0.97 |  |
|  | 25 | 1.00 | 0.90 | 0.93 | 0.97 |  |
|  | 50 | 0.88 | 0.79 | 0.91 | 0.96 |  |
|  | 100 | 0.89 | 0.85 | 0.94 | 0.97 |  |
|  | 150 | 0.87 | 0.85 | 0.94 | 0.98 |  |
|  | MRZ (nM) |  |  |  |  |  |
| **MAF-1337A** | | 10 | 20 | 50 | 75 | CQ (uM) |
|  | 10 | 1.07 | 1.11 | 1.03 | 1.01 |  |
|  | 25 | 1.02 | 1.08 | 1.05 | 1.02 |  |
|  | 50 | 1.00 | 1.00 | 1.02 | 1.01 |  |
|  | 100 | 1.00 | 1.00 | 1.01 | 1.00 |  |
|  | 150 | 0.99 | 0.99 | 1.01 | 1.00 |  |
|  | MRZ (nM) |  |  |  |  |  |
| **MAF-737A** |  | 10 | 20 | 50 | 75 | CQ (uM) |
|  | 5 | 1.03 | 1.04 | 1.00 | 1.00 |  |
|  | 10 | 1.03 | 1.10 | 1.00 | 1.00 |  |
|  | 25 | 1.18 | 1.07 | 0.99 | 0.99 |  |
|  | 50 | 1.18 | 1.04 | 0.96 | 0.99 |  |
|  | 100 | 1.08 | 0.98 | 0.95 | 0.98 |  |
|  | MRZ (nM) |  |  |  |  |  |
| **BT12** |  | 10 | 20 | 50 | 75 | CQ (uM) |
|  | 5 | 1.00 | 1.00 | 1.01 | 1.00 |  |
|  | 10 | 1.04 | 1.02 | 1.01 | 1.00 |  |
|  | 25 | 1.18 | 1.09 | 1.02 | 1.00 |  |
|  | 50 | 1.05 | 1.01 | 1.00 | 1.00 |  |
|  | 100 | 1.02 | 1.00 | 1.00 | 1.00 |  |
|  | MRZ (nM) |  |  |  |  |  |
| **BT16** |  | 10 | 20 | 50 | 75 | CQ (uM) |
|  | 5 | 1.18 | 1.10 | 1.03 | 1.02 |  |
|  | 10 | 1.35 | 1.22 | 1.09 | 1.04 |  |
|  | 25 | 1.08 | 1.09 | 1.06 | 1.03 |  |
|  | 50 | 1.02 | 1.03 | 1.02 | 1.01 |  |
|  | 100 | 1.01 | 1.01 | 0.99 | 0.99 |  |
|  | MRZ (nM) |  |  |  |  |  |
