# Supplementary material for: Proteasome inhibition as a therapeutic approach in atypical teratoid/rhabdoid tumors
Source: Neurooncol Adv. 2020 Apr 14;2(1):vdaa051. doi: 10.1093/noajnl/vdaa051 (PMC7236404; doi:10.1093/noajnl/vdaa051)
Supplement: vdaa051_suppl_Supplementary_Table_and_Figure_Legends [file vdaa051_suppl_supplementary_table_and_figure_legends.docx]

**Supplementary Figure Legends**

Figure S1: Cell viability (%) after 3 days treatment with MRZ 100 nM, Q-VD-OPh 20 uM, N-acetyl cysteine 5 mM, or a combination thereof.

Figure S2: (A-E) Cell confluence, as determined by phase microscopy via Incucyte ZOOM for three days growth under treatment with MRZ 100 nM, Q-VD-OPh 20 uM, N-acetyl cysteine 5 mM, or a combination thereof. (F) Endpoint confluence measures for cell lines measured over time in (A-E). *p=0.024

Figure S3: qPCR for SOD1 expression in five AT/RT cell lines after 24 hours treatment with 100 nM MRZ. Statistics shown are for 2-way ANOVA.

Figure S4: Western blot analysis of MAF-737A tumor xenografts extracted from the cerebellum post-sacrifice, blotting for total ubiquitin (top) and K48-linked ubiquitin (bottom). Xenografts are from mice treated twice weekly with either 200 ug/kg MRZ (+) or an equivalent volume of vehicle (-).

Figure S5: Western blot showing accumulation of ubiquitinated proteins for MAF-737A, BT12, and BT16 cell lines after 6 hrs treatment with 100nM MRZ. Upper blot shows total ubiquitin, lower GAPDH loading control.

Table S1: Cell viability after five days treatment with the NCI Approved Oncology Drugs panel, measured using CellTiter-Glo (Promega)

Table S2: Bliss synergy scores for cell lines treated with varying doses of MRZ and CQ for five days. Scores are calculated form cell viability, measured using CellTiter-Glo (Promega), and calculated using the (E_A_+E_B_-E_A_E_B_)/E_AB_ equation.
